# Antibiotics that target mitochondria effectively eradicate cancer stem cells, across multiple tumor types: Treating cancer like an infectious disease

**DOI:** 10.18632/oncotarget.3174

**Published:** 2015-01-22

**Authors:** Rebecca Lamb, Bela Ozsvari, Camilla L. Lisanti, Herbert B. Tanowitz, Anthony Howell, Ubaldo E. Martinez-Outschoorn, Federica Sotgia, Michael P. Lisanti

**Affiliations:** ^1^ The Breakthrough Breast Cancer Research Unit, Institute of Cancer Sciences, University of Manchester, UK; ^2^ The Manchester Centre for Cellular Metabolism (MCCM), Institute of Cancer Sciences, University of Manchester, UK; ^3^ The Moor Allerton Preparatory School, Didsbury, Manchester, UK; ^4^ Departments of Pathology and Medicine, The Albert Einstein College of Medicine, Bronx, NY, USA; ^5^ The Kimmel Cancer Center, Philadelphia, PA, USA

**Keywords:** mitochondria, mitochondrial biogenesis, cancer stem cells, tumor initiating cells, antibiotics

## Abstract

Here, we propose a new strategy for the treatment of early cancerous lesions and advanced metastatic disease, via the selective targeting of cancer stem cells (CSCs), a.k.a., tumor-initiating cells (TICs). We searched for a global phenotypic characteristic that was highly conserved among cancer stem cells, across multiple tumor types, to provide a mutation-independent approach to cancer therapy. This would allow us to target cancer stem cells, effectively treating cancer as a single disease of “stemness”, independently of the tumor tissue type. Using this approach, we identified a conserved phenotypic weak point – a strict dependence on mitochondrial biogenesis for the clonal expansion and survival of cancer stem cells. Interestingly, several classes of FDA-approved antibiotics inhibit mitochondrial biogenesis as a known “side-effect”, which could be harnessed instead as a “therapeutic effect”. Based on this analysis, we now show that 4-to-5 different classes of FDA-approved drugs can be used to eradicate cancer stem cells, in 12 different cancer cell lines, across 8 different tumor types (breast, DCIS, ovarian, prostate, lung, pancreatic, melanoma, and glioblastoma (brain)). These five classes of mitochondrially-targeted antibiotics include: the erythromycins, the tetracyclines, the glycylcyclines, an anti-parasitic drug, and chloramphenicol. Functional data are presented for one antibiotic in each drug class: azithromycin, doxycycline, tigecycline, pyrvinium pamoate, as well as chloramphenicol, as proof-of-concept. Importantly, many of these drugs are non-toxic for normal cells, likely reducing the side effects of anti-cancer therapy. Thus, we now propose to treat cancer like an infectious disease, by repurposing FDA-approved antibiotics for anti-cancer therapy, across multiple tumor types. These drug classes should also be considered for prevention studies, specifically focused on the prevention of tumor recurrence and distant metastasis. Finally, recent clinical trials with doxycycline and azithromycin (intended to target cancer-associated infections, but not cancer cells) have already shown positive therapeutic effects in cancer patients, although their ability to eradicate cancer stem cells was not yet appreciated.

## INTRODUCTION

Next generation sequencing and many other very sophisticated means of mutational analysis have given us an incredibly detailed view, or “molecular portrait”, of the diversity of genetic modifications that occur during the development of human cancers [1-5].

Despite this knowledge of the genomic landscape of cancer, it still remains extremely difficult to identify what are the primary “driver-mutations”, in the context of a “sea” of many other genetic changes [1-5]. The emerging picture is that while a few driver-mutations are common to certain specific cancer sub-types, each patient's tumor is fairly unique in its complexity of genetic changes and that several divergent cancer cell clones may also co-exist, within a single tumor [1-5].

This enormous level of detail and genetic complexity makes it extremely difficult to design new diagnostics and targeted-therapeutics, to achieve the goals of personalized medicine. There are numerous examples in clinical practice of how targeting the suspected “driver mutations” has been disappointing. Cancer control is frequently short lived, even when these drugs are proven to be effective. For example, the BRAF inhibitor vemurafenib is approved for the treatment of patients whose melanoma harbors the V600E mutation, which is thought to be a driver mutation [6, 7]. However, after treatment with vemurafenib, cancer progression occurs within six months in the vast majority of these patients with V600E mutations [6, 7].

Instead, an alternative approach would be to focus on what is common between different tumor types, rather than on what is divergent between different cancers. One common tractable target may be the property of “stemness” in cancer cells.

Recently, Tomasetti and Vogelstein showed that the life-time risk of two-thirds of cancers could simply be accounted for by the number of times that a given tissue's stem cells undergo cell division [8]. This is consistent with the idea that during aging, somatic mutations may accumulate in tissue stem cells, driving the formation of cancer stem cells [8]. They further concluded that these somatic mutations accounted for more cancer cases than either inherited genetic disease or specific environmental risk factors. These observations are also consistent with the idea that cancer is essentially a disease of “stemness” gone awry [7, 8].

Based on this rather simple premise, using unbiased quantitative proteomic profiling [9], we have focused on identifying a global phenotypic property of cancer stem cells (CSCs) that could be targeted across multiple tumor types. We have identified this property as a strict dependence on mitochondrial biogenesis, for the anchorage-independent clonal expansion and survival of the CSC population.

Here, we show that 4-to-5 different classes of FDA-approved antibiotics, which inhibit mitochondrial biogenesis as an “off-target” effect, can be used to eradicate cancer stem cells, in 12 different cancer cell lines, across 8 different tumor types (breast, DCIS, ovarian, prostate, lung, pancreatic, melanoma, and glioblastoma (brain)). Thus, future clinical trials for testing the efficacy of these mitochondrially-targeted antibiotics, in multiple cancer types, are now clearly clinically warranted. Overall, the use of generic antibiotics for anti-cancer therapy should significantly reduce the costs of patient care, making treatment more accessible in the developing world.

## RESULTS

### Overall approach: Finding an Achilles' heel in cancer stem cells

Recently, we used an unbiased proteomics approach to identify what makes cancer stem cells relatively unique, as compared with ‘bulk’ cancer cells [9]. For this purpose, we characterized the proteome of mammo-spheres derived from two different ER(+) breast cancer lines, specifically MCF7 and T47D cells [9]. Interestingly, we observed that MCF7 mammo-spheres show the marked over-expression of >60 mitochondrial-related proteins, as compared with monolayers; nine of these mitochondrial proteins were infinitely upregulated in mammo-spheres [9]. These findings are consistent with the idea that cancer stem cells are anabolic and that they may require mitochondrial biogenesis for their survival and proliferative expansion [9].

To test this hypothesis more directly, here we took advantage of the known side effects of specific classes of antibiotics. Because mitochondria evolved from bacteria that were originally engulfed by eukaryotic cells millions of years ago (known as the “endosymbiotic theory of mitochondrial evolution”) [10, 11], many classes of FDA-approved antibiotics actually target mitochondria, as a mild side-effect, which is well-tolerated in most patients.

**Figure 1:**
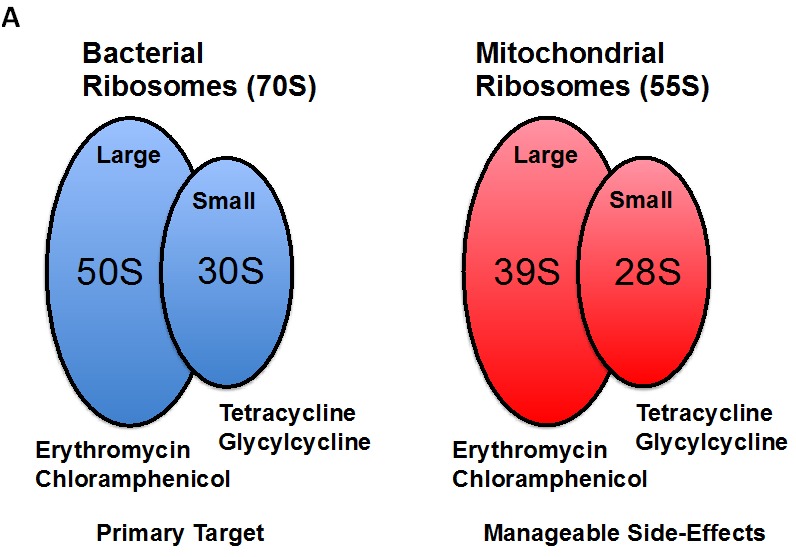

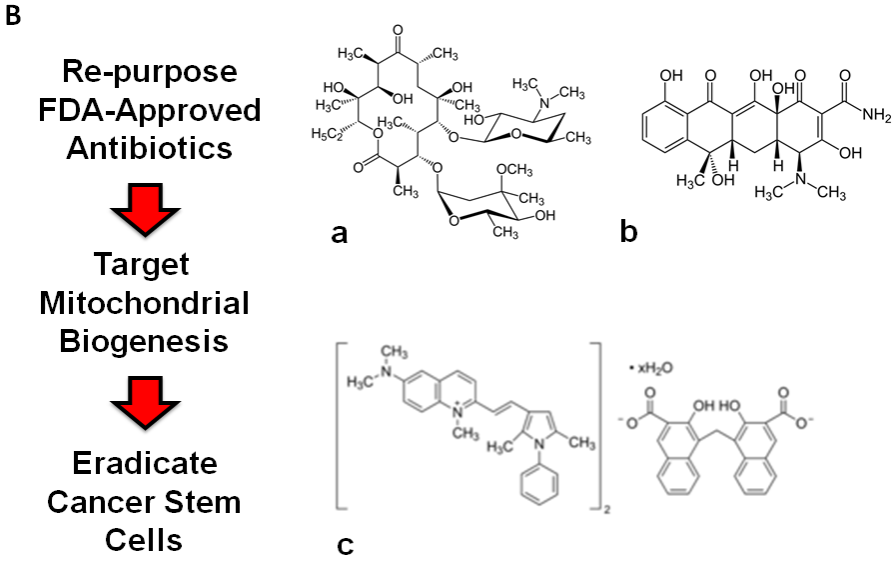
Treating cancer like an infectious disease, with antibiotics Recently, using unbiased proteomics analysis, we showed that mitochondrial proteins were highly upregulated in MCF7 and T47D tumor-spheres, as directly compared with monolayer cells. Thus, we set out to test the hypothesis that tumor-sphere formation was strictly dependent on mitochondrial biogenesis. Interestingly, several known classes of FDA-approved antibiotics function as inhibitors of mitochondrial biogenesis, which results in manageable side-effects. (A) Bacterial and mitochondrial ribosomes are closely related. Erythromycins and chloramphenicol target the large mitochondrial ribosome, while tetracyclines and glycylcyclines target the small mitochondrial ribosomes, because of conserved similarities with bacterial ribosomes. (B) Examples of FDA-approved antibiotics are shown. The structures of erythromycin (a) and tetracycline (b) are shown, along with pyrvinium pamoate (c). Here, we tested the hypothesis that these different classes of FDA-approved antibiotics could be re-purposed for the targeting of mitochondrial biogenesis and the eradication of cancer stem cells.

More specifically, the erythromycins and chloramphenicol selectively bind to the large subunit of the mitochondrial ribosome and inhibit mitochondrial biogenesis, by preventing the translation of mitochondrial proteins, mainly related to the mitochondrial OXPHOS complexes (Figure 1A). Similarly, the tetracyclines and glycylcyclines both bind with high affinity to the small subunit of the mitochondrial ribosome and inhibit mitochondrial biogenesis as well (Figure 1A).

Thus, these four large classes of antibiotics all function as known inhibitors of mitochondrial biogenesis in mammalian cells. This would allow us to use these antibiotics as “investigational tools” to assess if mitochondrial biogenesis is absolutely required for the survival and propagation of cancer stem cells. If successful, these FDA-approved antibiotics could then be re-purposed for the treatment of cancer, to achieve the eradication or more effective elimination of cancer stem cells (Figure 1B).

**Figure 2:**
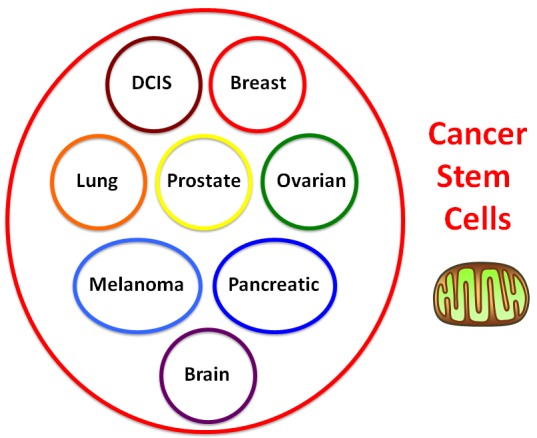
Treating cancer phenotypically as a single disease of increased “stemness”: Developing a mutation-independent approach to cancer therapy One idea is that we could potentially treat cancer as a single disease, if we could identify a global phenotypic characteristic that is conserved across multiple cancer types. For this purpose, we focused on tumor-initiating cells (TICs), a.k.a., cancer stem cells (CSCs), as they share many common properties, such as anchorage-independent growth, i.e., tumor-sphere formation under low-attachment conditions. This global phenotypic property appears to be functionally dependent on increased mitochondrial biogenesis.

If mitochondrial biogenesis is indeed required for the propagation of all cancer stem cells, then this new therapeutic approach could be applied across multiple cancer types, perhaps in a mutation independent fashion. In essence, we would be treating cancer based instead on a common global phenotypic property that is characteristic of cancer stem cells, allowing this approach to be more broadly applied perhaps to any cancer type (Figure 2).

### The Erythromycins: Azithromycin as an example

Azithromycin is a macrolide antibiotic that is currently used for the treatment of many types of bacterial infections. Azithromycin is a derivative of erythromycin, and is generally more potent and is more slowly eliminated than erythromycin, allowing infections to be treated relatively quickly, over 3-to-5 days. Overall, it shows broad-spectrum anti-bacterial activity. Mechanistically, azithromycin inhibits bacterial growth by preventing protein synthesis. Azithromycin directly binds to the 50S subunit of the bacterial ribosome, and specifically inhibits the translation of mRNA species into protein. Importantly, the 50S bacterial ribosome is homologous the 39S mitochondrial ribosome. In fact, many of the bacterial ribosomal subunits have directly-related mitochondrial homologues in eukaryotic cells. As such, erythromycin-related antibiotics, e.g., azithromycin, behave as inhibitors of mitochondrial biogenesis.

**Figure 3:**
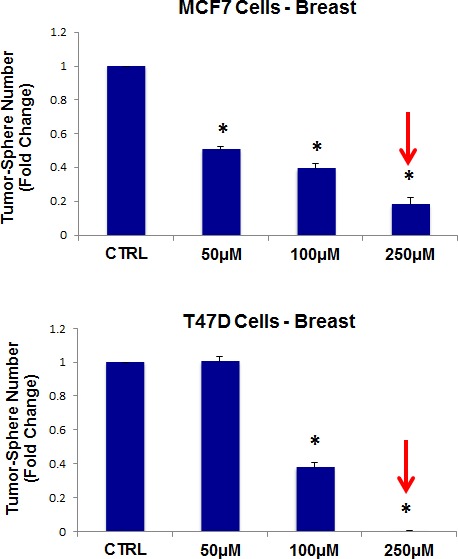
Azithromycin dose-dependently inhibits tumor-sphere formation in MCF7 and T47D cells, two commonly used ER(+) breast cancer cell lines Azithromycin was initially tested over the range of 50 μM to 250 μM. Note that 250 μM was the most effective. (*)p <0.001.

Thus, as a first step, we chose to evaluate the efficacy of azithromycin. For this purpose, azithromycin was tested for its ability to inhibit mammo-sphere formation using MCF7 and T47D cells, over a range of concentrations. Interestingly, in MCF7 cells, azithromycin inhibited mammo-sphere formation with an IC-50 of ~50 μM; similar results were also obtained in T47D cells (Figure 3).

**Figure 4:**
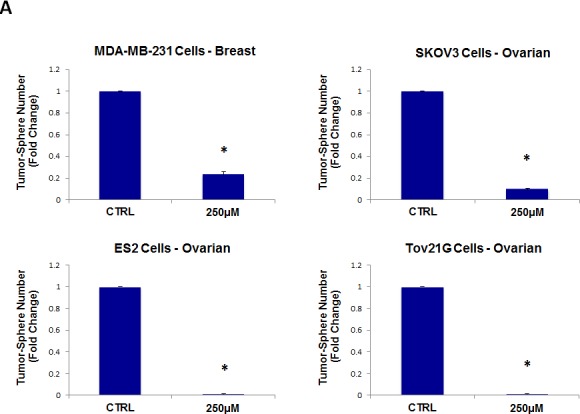

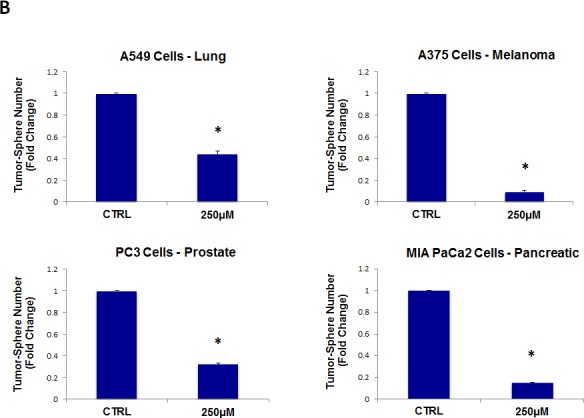
Azithromycin inhibits tumor-sphere formation in eight other cell lines, derived from diverse cancer types For simplicity, the efficacy of azithromycin was tested at a concentration of 250 μM. (*)p <0.001. (A) ER(−) breast [MDA-MB-231] and ovarian cancer cell lines [SKOV3, ES2, Tov21G] (B) Lung [A>549], prostate [PC3], melanoma [A375], and pancreatic [MIA PaCa2].

As a consequence of these positive findings, we next tested the ability of azithromycin to inhibit tumor-sphere formation in a wide-variety of cell lines derived from many different tumor types, including ER(−) breast cancer, ovarian, lung, pancreatic, and prostate cancer, as well as melanoma (summarized in Table 1). For simplicity, we used a single concentration of 250 μM. Figure 4 (panels A and B) directly shows that azithromycin inhibited tumor-sphere formation in these 8 additional well-established cell lines, representing 6 different cancer types. Thus, azithromycin was effective against tumor-sphere formation in all 10 cell lines tested.

**Table 1:** Twelve Cancer Cell Models with Broad Applicability

Cancer Types	Cell Lines
**Breast (ER+)**	MCF7
	T47D
**Breast (ER-)**	MDA-MB-231
**DCIS**	MCFIO.DCIS.com (“pre-malignant”)
**Ovarian**	SKOV3
	Tov2lG
	ES2
**Prostate**	PC3
**Pancreatic**	MIA PaCa2
**Lung**	A549
**Melanoma**	A375
**Glioblastoma**	U-87 MG

12 Cell Lines Representing 8 Different Types of Cancer.

### The Tetracyclines: Doxycycline provides proof-of-concept

Tetracycline is a broad-spectrum antibiotic that is commonly used for the treatment many bacterial infections, and functions as an inhibitor of protein synthesis in bacteria. Today, it is mainly used for the treatment of acne. However, the term “tetracyclines” is also used to describe a class of related semi-synthetic derivatives, that all contain the same chemically-conserved four-member ring structure, that is characteristic of tetracycline. Tetracyclines show bacterio-static activity against nearly all aerobic and anaerobic bacteria, including both Gram-positive and Gram-negative types. Tetracyclines inhibit protein synthesis by preventing the binding of activated aminoacyl-tRNAs to the A-site on the 30S subunit of bacterial ribosomes. As such, they reversibly inhibit the addition of new amino acids to the growing polypeptide chain, during protein synthesis. Importantly, the 30S bacterial ribosome is homologous the 28S mitochondrial ribosome, accounting for the manageable side-effects of the tetracyclines. As a consequence, tetracycline-based antibiotics are inhibitors of mitochondrial biogenesis. Doxycycline is a tetracycline-derivative with markedly improved efficacy and stability, which was first FDA-approved in the late 1960s, nearly 50 years ago now.

**Figure 5:**
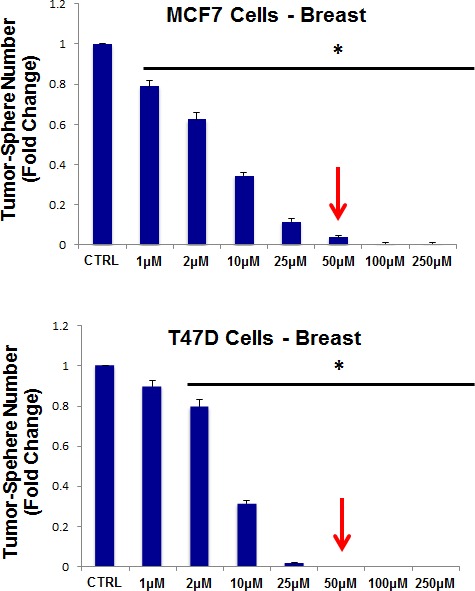
Doxycycline dose-dependently inhibits tumor-sphere formation in MCF7 and T47D cells, two commonly used ER(+) breast cancer cell lines Doxcycline was initially tested over the range of 1 μM to 250 μM. Note that 50-to-100 μM was the most effective. (*)p <0.001.

Thus, we next evaluated the efficacy of the tetracycline-based antibiotic, doxycycline. More specifically, doxycycline was tested for its ability to inhibit mammo-sphere formation using MCF7 and T47D cells, over a large range of concentrations, from 1 μM to 250 μM. Interestingly, in MCF7 cells, doxycycline inhibited mammo-sphere formation with an IC-50 between 2 and 10 μM; virtually identical results were also obtained in T47D cells (Figure 5).

**Figure 6:**
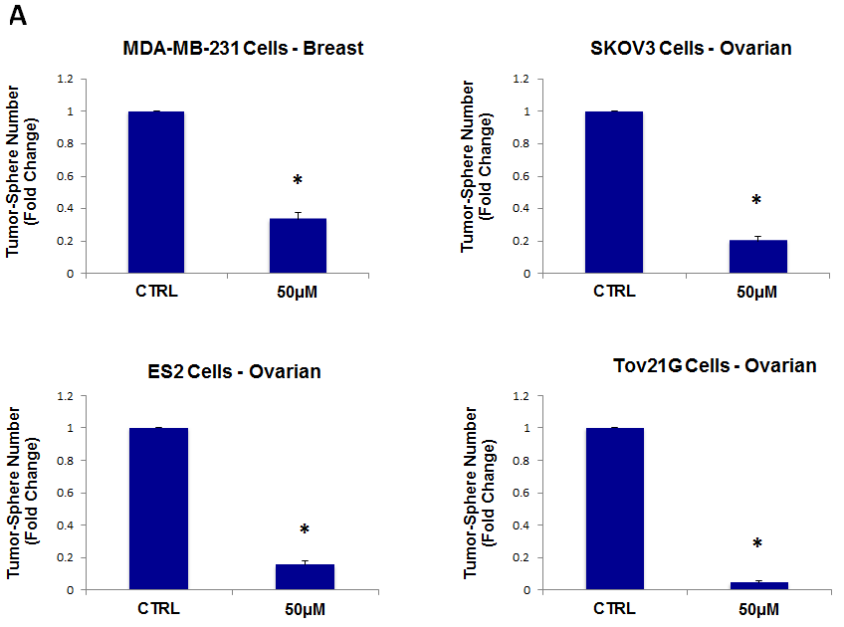

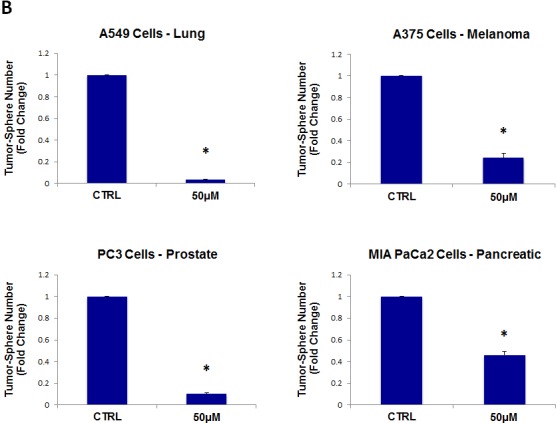
Doxycycline inhibits tumor-sphere formation in eight other cell lines, derived from diverse cancer types For simplicity, the efficacy of doxycycline was tested at a concentration of 50 μM. (*)p <0.001. (A) ER(−) breast [MDA-MB-231] and ovarian cancer cell lines [SKOV3, ES2, Tov21G] (B) Lung [A549], prostate [PC3], melanoma [A375], and pancreatic [MIA PaCa2].

We then tested the ability of doxycycline to inhibit tumor-sphere formation in a broad panel of cancer cell lines derived from many different tumor types (Table 1). For simplicity, we used a single concentration of 50 μM. Figure 6 (panels A and B) directly shows that doxycycline inhibited tumor-sphere formation in all of these well-established cell lines. Thus, doxycycline was effective against tumor-sphere formation in all 10 cell lines tested, across 6 different cancer types.

### The Glycylcyclines: Tigecycline shows efficacy

The glycylcyclines are a relatively new antibiotic class that are highly-related to the tetracyclines. The glycylcyclines were designed to specifically overcome tetracycline resistance. They have essentially the same mechanism of action as the tetracyclines, as they prevent bacterial protein synthesis. Both tetracyclines and glycylcyclines bind to the 30S bacterial ribosomal subunit, thereby inhibiting the binding of a given aminoacyl-tRNA to the A-site of the ribosome. Importantly, it appears that glycylcyclines bind more tightly to the ribosome, than the tetracyclines. Currently, tigecycline is the only FDA-approved glycylcycline. Similarly, the glycylcyclines are inhibitors of mitochondrial biogenesis.

**Figure 7:**
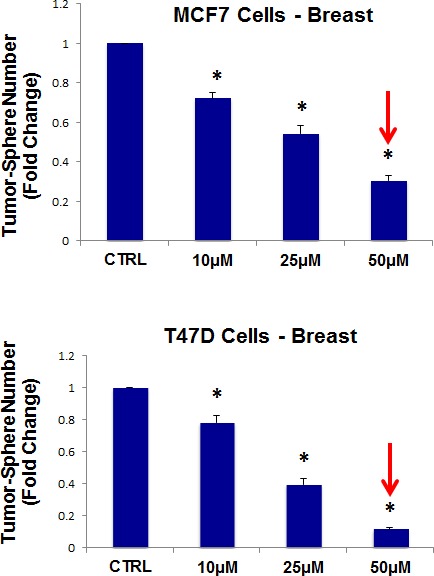
Tigecycline dose-dependently inhibits tumor-sphere formation in MCF7 and T47D cells, two commonly used ER(+) breast cancer cell lines Tigecycline was initially tested over the range of 10 μM to 50 μM. Note that 50 μM was the most effective. (*)p <0.001.

**Figure 8:**
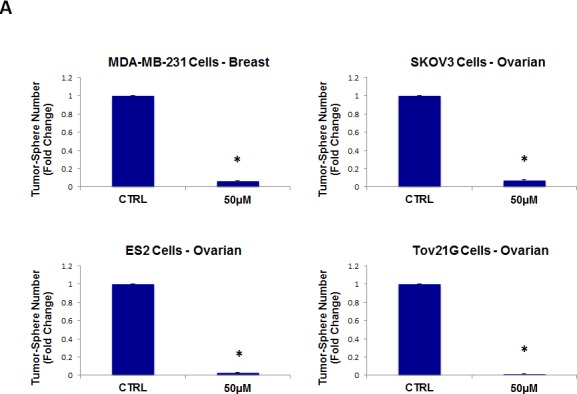

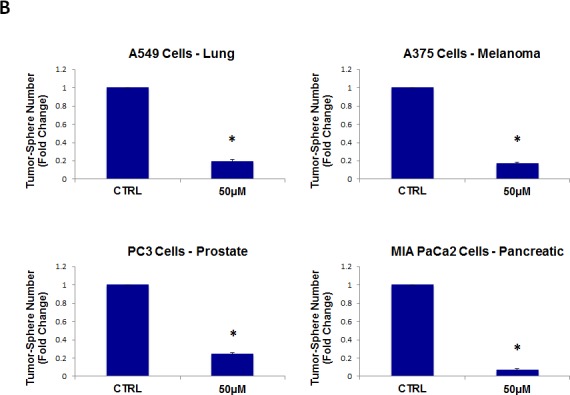
Tigecycline inhibits tumor-sphere formation in eight other cell lines, derived from diverse cancer types For simplicity, the efficacy of tigecycline was tested at a concentration of 50 μM. (*)p <0.001. (A) ER(−) breast [MDA-MB-231] and ovarian cancer cell lines [SKOV3, ES2, Tov21G] (B) Lung [A549], prostate [PC3], melanoma [A375], and pancreatic [MIA PaCa2].

Interestingly, quantitatively similar results were obtained with tigecycline, as compared with doxycycline, showing that it also has the capacity to inhibit tumor-sphere formation, across all 10 cell lines tested. Tigecycline was first tested for its ability to inhibit mammo-sphere formation using MCF7 and T47D cells, over a concentration range, from 10 μM to 50 μM. Interestingly, in both MCF7 and T47D cells, tigecycline inhibited mammo-sphere formation with an IC-50 between 10 and 25 μM (Figure 7). For simplicity, we used a single concentration of 50 μM, in all eight other cell lines, which significantly inhibited tumor-sphere formation (Figures 8).

### Anti-parasitc drugs: Pyrvinium pamoate

Pyrvinium is a cyanine dye, which is an FDA-approved anti-helmintic drug, that has been used to treat pinworms, as well as strongyloidiasis in humans. It was first approved by the FDA in 1955 for the treatment of enterobiasis, and is known to act as an inhibitor of mitochondrial oxidative phosphorylation (OXPHOS), under both normoxia and hypoxic conditions. Several forms of pyrvinium have been prepared, with different anions. Here, we tested the efficacy of pyrvinium pamoate. We used this approach to further validate that mitochondrial function was indeed critical for the survival and propagation of cancer stem cells.

**Figure 9:**
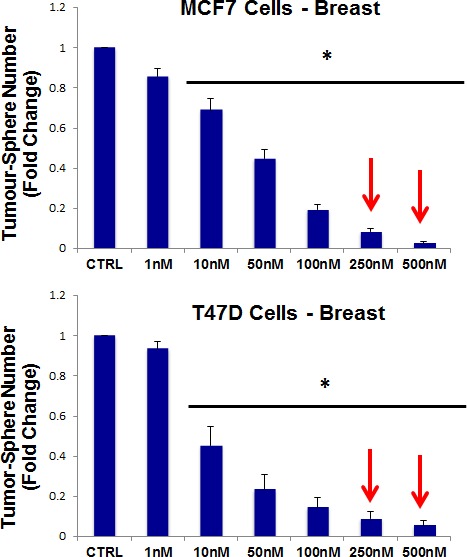
Pyrvinium pamoate dose-dependently inhibits tumor-sphere formation in MCF7 and T47D cells, two commonly used ER(+) breast cancer cell lines Pyrvinium pamoate was initially tested over the range of 1 nM to 500 nM. Note that 250 nM and 500 nM were the most effective. (*)p <0.001.

For this purpose, pyrvinium pamoate was tested for its ability to inhibit mammo-sphere formation using MCF7 and T47D cells, over a range of concentrations, from 1 nM to 500 nM. Interestingly, in both MCF7 and T47D cells, pyrvinium pamoate inhibited mammo-sphere formation with an IC-50 between ~10-to-50 nM (Figure 9).

**Figure 10:**
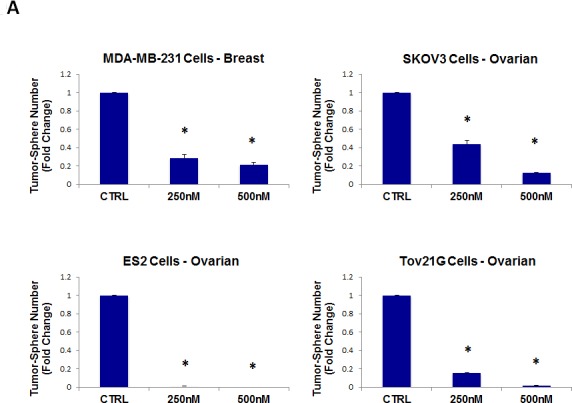

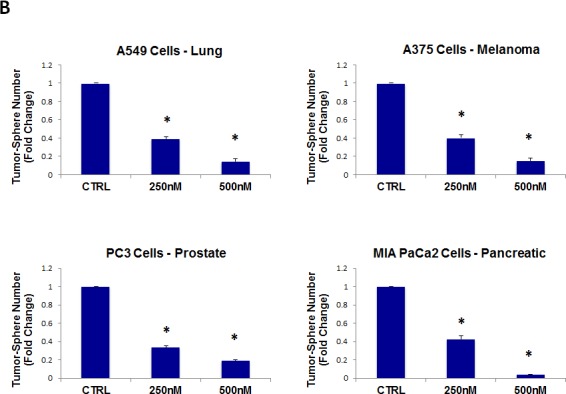
Pyrvinium pamoate inhibits tumor-sphere formation in eight other cell lines, derived from diverse cancer types For simplicity, the efficacy of pyrvinium pamoate was tested at a concentration of 250 nM and 500 nM. (*)p <0.001. (A) ER(−) breast [MDA-MB-231] and ovarian cancer cell lines [SKOV3, ES2, Tov21G] (B) Lung [A549], prostate [PC3], melanoma [A375], and pancreatic [MIA PaCa2].

As such, we next tested the ability of pyrvinium pamoate to inhibit tumor-sphere formation in a wide-variety of cell lines derived from many different tumor types (listed in Table 1); we used two concentrations of 250 nM and 500 nM. Figure 10 (panels A and B) illustrates that pyrvinium pamoate inhibited tumor-sphere formation across the entire cell line panel; pyrvinium pamoate was effective against tumor-sphere formation in all 10 cell lines tested, in the nano-molar range.

### Efficacy of four classes of antibiotics against DCIS and Glioblastoma CSCs

To further assess the efficacy of these four classes of antibiotics, we also determined their effectiveness in inhibiting tumor-sphere formation using two other well-established cell lines. One was a DCIS-based human cell line, namely MCF10.DCIS.com, which is often used to model pre-malignant mammary lesions in breast cancer patients. The other was derived from one of the most malignant and aggressive tumor types, glioblastoma (brain cancer; U-87 MG).

**Figure 11:**
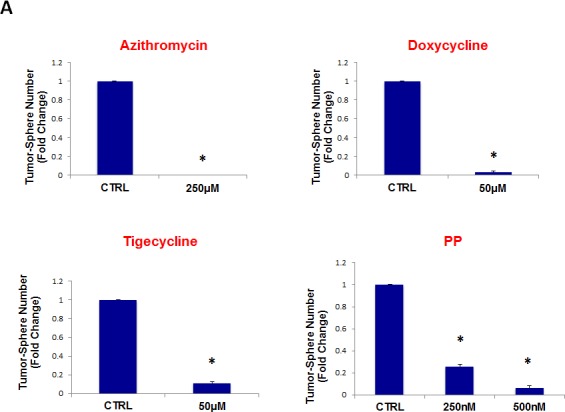

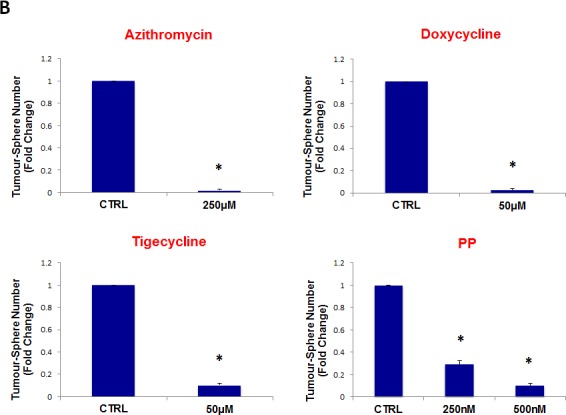
Four FDA-approved antibiotic classes also inhibit tumor-sphere formation in DCIS and glioblastoma cell lines Antibiotics were tested at the following concentrations: azithromycin (250 μM), doxycycline (50 μM), tigecycline (50 μM), and pyrvinium pamoate (250 and 500 nM). Note that all four antibiotics classes inhibit tumor-sphere formation, in both DCIS and glioblastoma cell lines. (A) DCIS.com; (B) U-87 MG. (*)p <0.001.

Figure 11 (panels A and B) shows that all four classes of antibiotics significantly inhibited tumor-sphere formation in both DCIS.com and U-87 MG cells. Thus, our approach can also be used to target both pre-malignant lesions, such as DCIS, or even the most invasive and aggressive cancer types, such as glioblastoma.

Importantly, azithromycin, doxycycline, and tigecycline are all known to cross the blood-brain barrier, making the treatment of brain cancer with these antibiotics feasible.

### Evaluating possible toxicity in “bulk” cancer cells and normal fibroblasts

To evaluate the possible toxicity of our approach on ”bulk” cancer cells and normal fibroblasts, we next used i) MCF7 cell monolayers and ii) hTERT-immortalized skin fibroblasts, and assessed their viability using the MTS assay. For these experiments, we focused on two drugs, namely doxycycline and pyrvinium pamoate.

**Figure 12:**
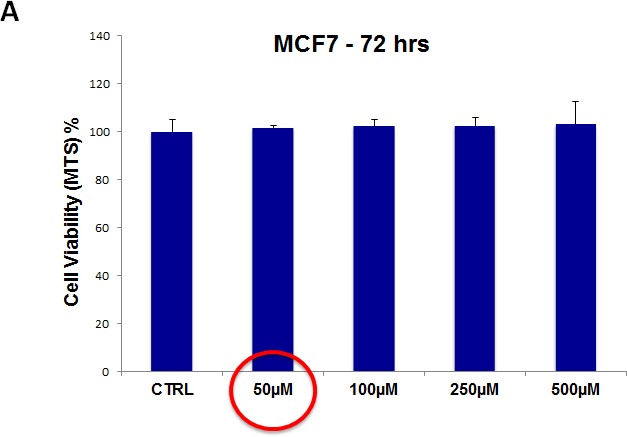

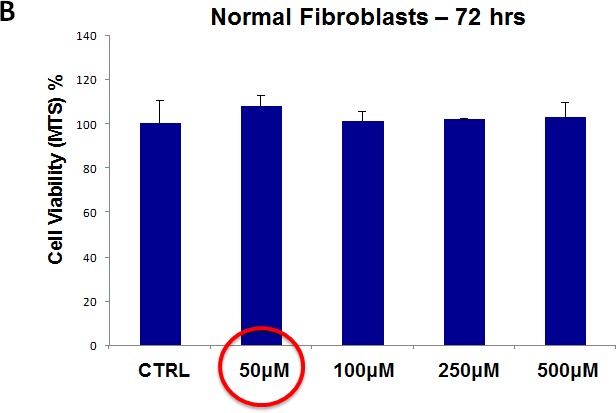
Doxycycline does not affect the viability of “bulk” cancer cells or normal fibroblasts (A) MCF7 cell monolayers; (B) hTERT-BJ1 skin fibroblasts. Viability was quantitatively measured using the MTS assay. Doxcycline was tested over the range of 50 μM to 500 μM, with little or no effects on cell viability.

For doxycycline, we used a concentration range from 50 μM to 500 μM. Note that there was little or no toxicity observed in MCF7 cell monolayers or hTERT-BJ1 fibroblasts, over this entire range (Figure 12, panels A and B). Importantly, 50 μM doxycycline reduced MCF7 mammosphere formation by >95% (Figure 5). Thus, there is no toxicity for MCF7 cell monolayers or normal fibroblasts at a concentration that nearly completely eliminates CSC expansion (and even at 10X times higher).

**Figure 13:**
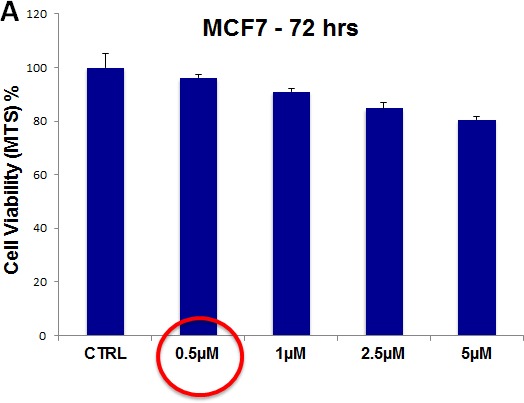

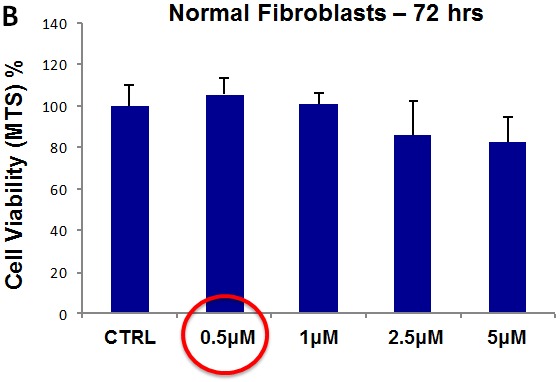
Pyrvinium pamoate does not affect the viability of “bulk” cancer cells or normal fibroblasts (A) MCF7 cell monolayers; (B) hTERT-BJ1 skin fibroblasts. Viability was quantitatively measured using the MTS assay. Pyrvinium pamoate was tested over the range of 500 nM to 5 μM, with little or no effects on cell viability.

For pyrvinium pamoate, we used a concentration range from 500 nM to 5 μM. Importantly, there was little or no toxicity observed in MCF7 cell monolayers or hTERT-BJ1 fibroblasts (Figure 13, panels A and B). Interestingly, 500 nM pyrvinium pamoate reduced MCF7 mammosphere formation by >99% (Figure 9). As such, there is little or no toxicity for MCF7 cell monolayers or normal fibroblasts at a concentration that eliminates CSC expansion (and even at 10X times higher).

Importantly, we observed very limited toxicity, which is consistent with fact that these are well-tolerated antibiotics that are already FDA-approved for patient therapy, but are normally used in the context of infectious disease.

### Chloramphenicol

Chloramphenicol is a bacterio-static antibiotic used for the treatment of a number of bacterial infections, which first became available in the late 1940s. It is a typical broad-spectrum antibiotic. Like the eyrthromycins and the tetracyclines, it functions as an inhibitor of protein synthesis, by binding to the 50S bacterial ribosomal subunit, thereby inhibiting peptide bond formation. More specifically, it inhibits peptidyl-transferase activity, preventing protein chain elongation. Similarly, in mammalian cells, chloramphenicol behaves an inhibitor of mitochondrial biogenesis.

**Figure 14:**
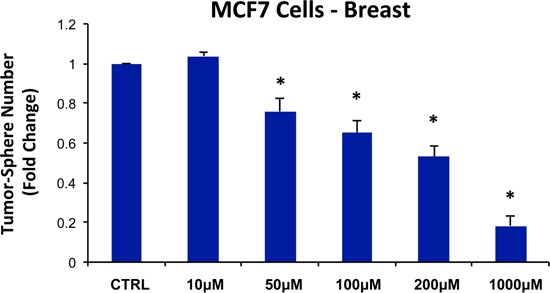
Chloramphenicol dose-dependently inhibits tumor-sphere formation in MCF7 cells We determined the ability of chloramphenicol to inhibit mammo-sphere formation using MCF7 cells, over a range of concentrations from 10 μM to 1 mM. Interestingly, in MCF7 cells, chloramphenicol inhibited mammo-sphere formation with an IC-50 of ~200 μM. (*)p <0.002.

Thus, we chose to evaluate the efficacy of chloramphenicol and determined its ability to inhibit mammo-sphere formation using MCF7 cells, over a range of concentrations from 10 μM to 1 mM. Interestingly, in MCF7 cells, chloramphenicol inhibited mammo-sphere formation with an IC-50 of ~200 μM (Figure 14). As such, we have now shown that four independent antibiotic inhibitors of mitochondrial biogenesis (azithromycin, doxycycline, tigecycline, and chloramphenicol) all effectively inhibit mammo-sphere formation. However, chloramphenicol was the least potent of the mitochondrial inhibitors that we tested.

## DISCUSSION

**Figure 15:**
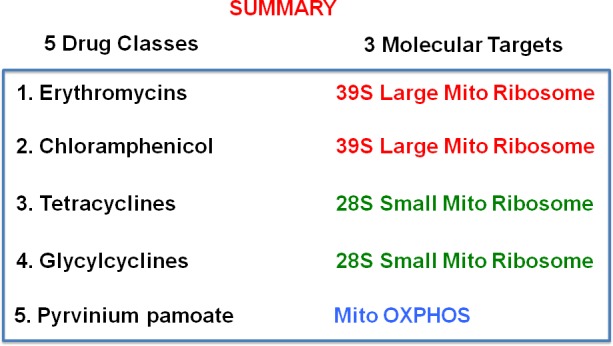
Summary of drug discovery The five-classes of antibiotics that we tested are summarized here, along with their three corresponding molecular targets, which all converge on mitochondrial biogenesis or OXPHOS.

Here, we showed that 4-to-5 different classes of FDA-approved antibiotics can be used to selectively target CSCs, across multiple tumor types. Mechanistically, these antibiotics converge on three main mitochondrial targets, as summarized in Figure 15. Thus, molecular disruption of mitochondrial biogenesis or OXPHOS would be a novel therapeutic strategy for the eradication of CSCs. As a result, our findings have broad implications for the initiation of new clinical trials, for the re-purposing of antibiotics for the treatment of various cancer types, including “pre-malignant” and advanced metastatic disease.

This new therapeutic strategy takes advantage of the manageable side-effects of these antibiotics, which affect eukaryotic mitochondria, although these compounds are currently used for the broad-spectrum treatment of bacterial and parasitic infectious diseases.

In this regard, doxycycline is relatively attractive as a new anti-cancer agent, as it has a long half-life systemically and has been used successfully for the long-term treatment of patients with urinary tract infections (UTI), prostatitis or acne, for extended periods of time, of up to 4-to-6 months or more (200 mg per day). Doxycycline also encourages the growth of normal stem cells, has anti-inflammatory properties, and even increases lifespan, in certain experimental contexts [12-14]. Thus, the toxic side effects of anti-cancer therapy would be minimized.

Doxycycline has also been used in human tumor xenografts and other animal models to significantly reduce tumor burden and even metastatic cancer cell growth [15-20]. For example, in pancreatic tumor xenografts (with PANC-1 cells), doxycycline treatment reduced tumor growth by ~80% [20]. In a xenograft model of breast cancer bone metastasis (with MDA-MB-231 cells), doxycycline treatment reduced bone and bone-associated soft-tissue tumor mass by >60% and ~80%, respectively [19]. However, its anti-cancer activity was attributed to the inhibition of matrix-metalloproteinases (MMPs), rather than the targeting of mitochondrial biogenesis, and doxycycline has not been previously implicated in the selective eradication of cancer stem cells [15-20].

Our results are consistent with the previous finding that metformin, a widely used anti-diabetic drug, which functions as a mitochondrial inhibitor, can also be used to selectively target CSCs [21, 22]. Metformin functionally inhibits OXPHOS by targeting complex I of the electron transport chain and can even induce lactic acidosis, as a lethal side effect [21, 22]. As a result, the use of antibiotics, such as doxycycline, may provide a safer and far more effective alternative to anti-cancer therapy with metformin.

**Figure 16:**
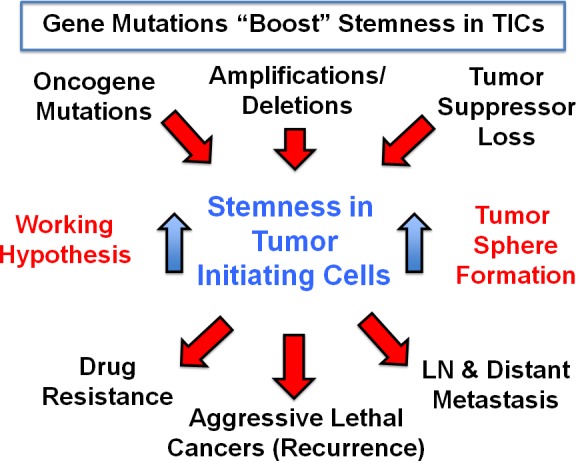
A mutation-independent approach to cancer therapy We speculate that genetic changes (oncogenic mutations, amplifications/deletions, and tumor suppressor loss) all converge on “stemness” in tumor-initiating cells, driving tumor recurrence, metastasis and drug resistance. Thus, it would be advantageous to phenotypically target “stemness” directly, instead of targeting individual genetic changes, in different cancer types. This would allow the treatment of cancer as a single disease of stemness, in a mutation-independent fashion.

Our global phenotypic approach to target cancer as a single disease of stemness, may also help to avoid drug resistance. We speculate that genetic changes (oncogenic mutations, amplifications/deletions, and tumor suppressor loss) all converge on “stemness” in tumor-initiating CSCs (Figure 16), driving tumor recurrence, metastasis and drug resistance. Thus, it would be advantageous to phenotypically target “stemness” directly, instead of targeting individual genetic changes, in different cancer types. This would allow the treatment of cancer in a mutation-independent fashion.

Finally, recent clinical trials with doxycycline and azithromycin (intended to treat cancer-associated infections, but not cancer cells) both show positive therapeutic effects in cancer patients, although their selective effects on eradicating cancer stem cells were not yet known or appreciated [23-26]. These trials were performed on advanced or treatment-resistant patients with B-cell lymphoma (doxycycline) or lung cancer (azithromycin), respectively [23-26]. For example, in lung cancers, azithromycin significantly increased 1-year patient survival from 45% to 75%, an ~1.7-fold increase [26]. Interestingly, it was noted that even lymphoma patients that were “bacteria-free” benefited from only a 3-week course of doxycycline therapy, and showed complete remission of the disease [27]. These results suggest that the antibiotic's therapeutic effects were actually infection-independent.

Thus, future clinical trials for testing the efficacy of mitochondrially-targetd antibiotics in multiple cancer types are now clearly clinically warranted. In this regard, a clinical trial with doxycycline in patients with advanced breast cancer and bone metastasis is ongoing: https://clinicaltrials.gov/ct2/show/NCT01847976. Secondly, a trial of doxycycline in relapsed patients with non-hodgkin's lymphoma has also been initiated: https://clinicaltrials.gov/ct2/show/NCT02086591. Interestingly, the A375 human melanoma cell line harbors the B-RAF(V600E) mutation and we showed that CSCs derived from this cell line are highly-sensitive to all four of the antibiotics that we tested (azithromcyin, doxycycline, tigecycline, and pyrvinium pamoate). As such, additional cellular studies, and new clinical trails in melanoma patients with B-RAF mutations, may be indicated, to explore the use of antibiotics.

## MATERIALS AND METHODS

### Materials

Cancer cell lines were purchased from the ATCC or other commercially available sources. Antibiotics were all obtained commercially from Sigma-Aldrich. Gibco-brand cell culture media (DMEM/F12) was purchased from Life Technologies.

### Tumor-sphere Culture

A single cell suspension was prepared using enzymatic (1x Trypsin-EDTA, Sigma Aldrich, #T3924), and manual disaggregation (25 gauge needle) to create a single cell suspension. Cells were plated at a density of 500 cells/cm2 in mammosphere medium (DMEM-F12/B27/20ng/ml EGF/PenStrep) in non-adherent conditions, in culture dishes coated with (2-hydroxyethylmethacrylate) (poly-HEMA, Sigma, #P3932) [28]. Cells were grown for 5 days and maintained in a humidified incubator at 37°C at an atmospheric pressure in 5% (v/v) carbon dioxide/air. After 5 days for culture, spheres >50 μm were counted using an eye piece graticule, and the percentage of cells plated which formed spheres was calculated and is referred to as percentage tumor-sphere formation (TSF), and was normalized to one (1 = 100 % TSF) [28]. All experiments were performed in triplicate, three times independently, such that each data point represents the average of 9 replicates.

For tumor-sphere assays, all cell line derived spheroids (MCF7, T47D, MDA-MB-231, DCIS.com, SKOV3, Tov21G, ES2, and A549) were counted at 5 days after plating, with the exception of PC3, A375, and MIA PaCa2 cells, which were counted at 3 days post-seeding. Finally, U-87 MG spheroids were counted at 7 days. All data shown are the mean +/− the standard error of the mean (SEM).
